# The Predictive Role of Cardiac Troponin in Non-cardiac Surgery: A Study in the Greek Population

**DOI:** 10.7759/cureus.25408

**Published:** 2022-05-27

**Authors:** Panagiota Manthou, Georgios Lioliousis, Anna Korobeli, Panagiotis Vasileiou, Georgios Fildisis

**Affiliations:** 1 Nursing School, National and Kapodistrian University of Athens, Athens, GRC; 2 Intensive Care Unit, First Department of Respiratory Medicine, Thoracic Diseases General Hospital Sotiria, "Sotiria" Hospital, National and Kapodistrian University of Athens, Athens, GRC; 3 School of Health Sciences, Department of Nursing, National and Kapodistrian University of Athens, "Agioi Anargyroi" General Hospital, Athens, GRC; 4 Emergency Department, Thoracic Diseases General Hospital Sotiria, Athens, GRC

**Keywords:** surgery, prognosis, cardiac troponin, myocardial infarction, risk

## Abstract

Introduction

The incidence of postoperative myocardial ischemia (POMI) remains uncertain and underdiagnosed despite significant morbidity and mortality rates.

Methods

This study included patients who underwent non-cardiac surgery. Troponin T (TnT) was measured on the first three postoperative days. The revised cardiac risk index, HAS-BLED (hypertension, abnormal renal/liver function, stroke, bleeding history or predisposition, labile international normalized ratio (INR), elderly, drugs/alcohol concomitantly) bleeding score, and CHA2DS2-VASc (congestive heart failure, hypertension, age ≥ 75 years, diabetes mellitus, stroke or transient ischemic attack (TIA), vascular disease, age 65 to 74 years, sex category) score were combined. The receiver operating characteristic (ROC) curve was used to estimate the discriminative ability of preoperative troponin for myocardial ischemia (MI).

Results

Of 105 patients with a mean age of 69.1 years, 32.4% had MI. Hypertension, diabetes mellitus, and dyslipidemia were the main risk factors. A ROC analysis indicated that a preoperative value of 17.2 pg/ml or higher of troponin was significantly associated with MI. Moreover, a higher CHA2DS2-VASc score was associated with POMI.

Conclusions

POMI is associated with high mortality and a long stay in the intensive care unit. Routine use of different scores before surgery can be very useful.

## Introduction

Postoperative myocardial infarction (POMI) is a serious complication associated with high costs and mortality rates in the intensive care unit (ICU). Despite efforts to prevent the occurrence of such events and identify risk factors, the incidence of POMI remains high [1].

However, no prophylactic treatment for POMI has yet been successfully identified. Because of the incidence and pathophysiology of POMI, including the type of infarct (Q wave vs. non-Q wave), studies have tended either to use frequent measurements of cardiac enzymes, such as troponin or brain natriuretic peptide (BNP), and electrocardiographs (ECGs) or diagnostic testing based on the development of clinical symptoms or signs [2]. The majority of studies suggest that MI occurs in the first 48 to 72 hours after surgery. Because symptoms such as chest pain are easily disguised by postoperative pain management, including opioids, the clinical course of POMI is mainly underdiagnosed [3].

Currently, high-risk patients and cardiovascular events are primarily predicted by means of a combination of detailed examinations during the preoperative period. This procedure is expensive and inconvenient and requires the time and expertise of medical staff. Therefore, it is essential to formulate a cost-effective, convenient, and accurate method for preoperative risk assessment, and the use of cardiac biomarkers has been proposed for this role.

In particular, cardiac biomarkers, such as BNP, cardiac troponins (cTN), and C-reactive protein (CRP), can play an important role not only preoperatively but also in the first hour after surgery, when patients are transferred to the ICU. In addition, the European Society of Cardiology and European Society of Anaesthesiology guidelines on preoperative cardiac risk assessment recommends that preoperative BNP or NT-proBNP (N-terminal pro-b-type natriuretic peptide) measurement should be considered in high-risk patients undergoing non-cardiac surgery [4]. Cardiovascular risk assessment in the preoperative period may improve patient prognosis. Recently, high-sensitivity cardiac troponin (hs-cTn) assays have been introduced into routine clinical care, allowing accurate detection of MI for the first time [5].

Based on the recommendations to screen high-risk patients undergoing non-cardiac surgery for POMI, our study sought to establish a simple clinical protocol to identify high-risk patients and improve postoperative care in the ICU, given the heavy burden on medical and nursing staff.

## Materials and methods

The ethics committee of the Department of Nursing of the National Kapodistrian University of Athens granted ethical approval for this study (20/06/2018-266), and all participants gave prior written informed consent. Based on the incidence of POMI in previous studies and the desired width of the confidence interval (CI) at a 95% confidence level, we aimed to include at least 100 consecutive patients who met the inclusion criteria.

Study design

We conducted a prospective study at the General Oncology Hospital, Athens, where patients requiring general surgery are treated. We investigated the incidence of postoperative severe cardiac events with ischemia screening and predicted such events with a combination of risk calculators.

Inclusion and exclusion criteria

**Figure 1:**
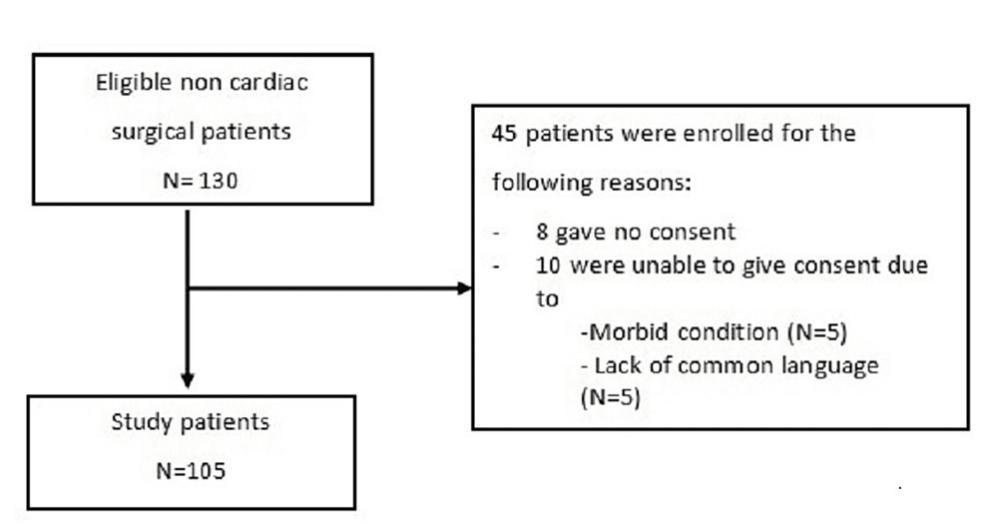
Patient flow

Between June 2018 and June 2021, all non-cardiac surgery patients between the ages of 18 and 85 undergoing non-cardiac surgical procedures who needed to stay in the ICU for more than 24 hours were eligible for the study. Ischemia monitoring was performed on each patient only after the first surgery. Written informed consent was obtained preoperatively from all eligible patients or their proxies. Patients (1) who refused consent, (2) who were unable to give consent, (3) whose consent could not be obtained for logistical/emergency reasons, or (4) who were diagnosed with MI before surgery were excluded. Patients with sepsis, pulmonary embolism, or chronic renal failure were excluded from the study. Also, patients with severe sepsis after surgery and pregnant women were excluded from the study. The postoperative follow-up to detect MI was conducted up to the first 72 hours after surgery until the patient was discharged from the ICU (Figure 1).

Definitions

The perioperative period was the time between the day before surgery and discharge from the hospital. The diagnosis of POMI was based on perioperative values and measurements of high-sensitivity troponin T (pg/ml) in combination with ischemic ECG changes or other ischaemic features such as chest pain, arrhythmias, and dyspnoea, or signs of MI on imaging. The diagnosis of POMI was made by a rise in TnT, with at least one value above the upper reference limit, and ischaemic ECG changes. Data on medications, including antithrombotic agents and statins, taken by the patient before and after PMI was also recorded. All patients with a positive troponin level after surgery were asked to see a cardiologist. Other medical conditions that could cause an increased TnT concentration were recorded and taken into account when making the diagnosis of MI. All patients who participated in the study were followed up for 30 days after surgery to assess their outcomes. Patients for whom no follow-up data were available were excluded from the final data analysis.

Data collection

Patient history and all medical and nursing data were obtained from the medical records. The first group, which was also the control group, includes patients who were admitted to the ICU for postoperative follow-up and did not have MI. The second group consisted of patients with MI in the first 72 hours postoperatively who required intensive care monitoring over a longer period of time. In addition, all patients with or without chronic disease were risk-assessed using the revised cardiac risk index (Cardiac Lee score), hemorrhage risk assessed using the HAS-BLED bleeding risk score, and thrombosis risk assessed using the CHA2DS2-VASc score. Laboratory tests, fluid balance, and transfusions were also assessed. A blood sample for TnT and other biomarkers, such as BNP, CRP, calcitonin, and an ECG, were systematically collected, preoperatively and postoperatively, in the first three days after surgery or until discharge. Sepsis was assessed by the sequential organ failure assessment (SOFA) system in both categories.

Statistical analyses

Quantitative variables were expressed as mean (standard deviation (SD)) or median (interquartile range (IQR)). Qualitative variables were expressed as absolute and relative frequencies. The chi-square and Fisher’s exact tests were used to compare proportions. When the normality assumption for the comparison of means between two groups was met, the student’s t-test was used. The Mann-Whitney test was used to compare continuous variables between two groups when the distribution was not normal. Receiver operating characteristic (ROC) curves were used to estimate the discriminatory ability of preoperative troponin for MI. Sensitivity, specificity, and negative and positive prognostic values were calculated for the optimal cut-off value. The area under the curve (AUC) was also calculated. Stepwise logistic regression analysis was performed (p for entry 0.05, p for exit 0.10) to find independent factors associated with MI. Adjusted odds ratios (OR) with 95% CI were calculated from the results of the logistic regression analyses. All reported p-values are two-sided values. Statistical significance was set at p < 0.05, and analyses were conducted using SPSS statistical software (Version 22.0; IBM Corp., Armonk, NY).

## Results

A total of 130 eligible patients underwent non-cardiac surgery. The sample consisted of 105 patients (53.3% men) with a mean age of 69.1 years (SD = 11.3 years). The baseline characteristics of the patients are listed in Table 1. Hypertension was present in 54.3% of the patients and diabetes mellitus in 24.8%. One in three patients had a history of heart disease, and 14.3% had had previous heart surgery. In addition, 32.4% of patients (N = 34) had MI. The use of antiplatelet drugs before surgery seems to play an important role in the occurrence of POMI (p < 0.001) while the timing of discontinuation of the corresponding drugs does not seem to play a role in the planned surgery (p = 0.818). One in three patients (33.3%) had a preoperative Lee score of 1, and 32.4% had a score of 2. However, a correlation of the results showed that the higher the Lee scores preoperatively, the higher the proportion of patients who developed POMI (p < 0.001). POMI was associated with an increased risk of death.

**Table 1:** Sample characteristics regarding clinical history COPD: chronic obstructive pulmonary disease; SOFA: sequential organ failure assessment; HAS-BLED: hypertension, abnormal renal/liver function, stroke, bleeding history or predisposition, labile international normalized ratio (INR), elderly, drugs/alcohol concomitantly; CHA2DS2-VASc: congestive heart failure, hypertension, age ≥ 75 years, diabetes mellitus, stroke or transient ischemic attack (TIA), vascular disease, age 65 to 74 years, sex category; IQR: interquartile range

	Ν (%)
Men	56 (53.3)
Women	49 (46.7)
Age, mean (SD)	69.1 (11.3)
Hypertension	57 (54.3)
Dyslipidemia	25 (23.8)
COPD	22 (21.0)
Diabetes mellitus	26 (24.8)
History of heart disease	35 (33.3)
Previous heart surgery	15 (14.3)
Antiplatelet therapy before surgery	40 (38.1)
ECG abnormalities before surgery	13 (12.4)
SOFA, mean (SD)	9.5 (3.4)
HAS-BLED score, mean (SD)	2.9 (1.2)
CHA2DS2-VASc score, mean (SD)	3 (1.5)
Blood transfusion during surgery	42 (40.0)
Pre-op. troponin, median (IQR)	12.3 (6.7 – 19.0)

Moreover, the percentage of POMI was significantly higher in patients with hypertension, dyslipidemia, COPD, diabetes mellitus, and a history of heart disease, as well as in patients who had had previous heart surgery. In addition, the percentage of MI was significantly higher in patients who systematically took antiplatelet drugs before surgery (p = 0.001) and in those who had preoperative ECG abnormalities. Preoperative troponin levels were significantly higher in patients with MI (p = 0.022) (Table 2).

**Table 2:** Myocardial ischemia association with patients’ characteristics +Pearson’s chi-square test; ++Fisher’s exact test; ‡Student’s t-test; ‡‡ Mann–Whitney test COPD: chronic obstructive pulmonary disease; SOFA: sequential organ failure assessment; HAS-BLED: hypertension, abnormal renal/liver function, stroke, bleeding history or predisposition, labile international normalized ratio (INR), elderly, drugs/alcohol concomitantly; CHA2DS2-VASc: congestive heart failure, hypertension, age ≥ 75 years, diabetes mellitus, stroke or transient ischemic attack (TIA), vascular disease, age 65 to 74 years, sex category; IQR: interquartile range

		Myocardial ischemia		
		No (N=71; 67.6%)	Yes (N=34; 32.4%)	
		Ν (%)	Ν (%)	P
Sex	Men	36 (50.7)	20 (58.8)	0.435+
	Women	35 (49.3)	14 (41.2)	
Age, mean (SD)		67.5 (11.8)	72.4 (9.7)	0.034‡
Hypertension	Νο	40 (83.3)	8 (16.7)	0.002+
	Yes	31 (54.4)	26 (45.6)	
Dyslipidemia	Νο	60 (75.0)	20 (25.0)	0.004+
	Yes	11 (44.0)	14 (56.0)	
COPD	Νο	60 (72.3)	23 (27.7)	0.047+
	Yes	11 (50)	11 (50)	
Diabetes	Νο	58 (73.4)	21 (26.6)	0.027+
	Yes	13 (50.0)	13 (50.0)	
History of heart disease	Νο	55 (78.6)	15 (21.4)	0.001+
	Yes	16 (45.7)	19 (54.3)	
Previous heart surgery	Νο	66 (73.3)	24 (26.7)	0.005++
	Yes	5 (33.3)	10 (66.7)	
Antiplatelet therapy	Νο	52 (80.0)	13 (20.0)	0.001+
	Yes	19 (47.5)	21 (52.5)	
Preoperative ECG abnormalities	Νο	66 (71.7)	26 (28.3)	0.025++
	Yes	5 (38.5)	8 (61.5)	
SOFA, mean (SD)		8.5 (3.1)	11.7 (2.9)	<0.001‡
HAS-BLED score, mean (SD)		2.7 (1.2)	3.2 (1.2)	0.050‡
CHA2, mean (SD)		2.5 (1.4)	3.9 (1.4)	<0.001‡
Blood transfusion during surgery	Νο	48 (76.2)	15 (23.8)	0.022+
	Yes	23 (54.8)	19 (45.2)	
Pre-op troponin, median (IQR)		12.0 (5.2 – 17.2)	17.3 (11.3 – 24.0)	0.022‡‡

The median length of hospital stay for all patients was 25 days (IQR: 15-35). Patients with POMI had a longer median hospital stay at 32 days (median range: 25-45) than the control group, which had a median duration of 18 days (median range: 14-30) (p < 0.001). Patients with MI also showed significant metabolic acidosis with pH < 7.25, lower HCO3, and significantly higher lactic acid, with a mean of 4.1 (p < 0.006). In addition, patients who required a transfusion of concentrated red blood cells or clotting factors in the first 24 hours postoperatively had a higher risk of developing MI in the first three days (p < 0.022).

The maximum dose of vasoconstrictor drugs received by patients with POMI was also significantly higher. In particular, they received almost three times higher doses of norepinephrine or a combination of two vasoactive drugs, such as norepinephrine and vasopressin, to achieve hemodynamic stability (p<0,001). Some patients also received an intravenous antiarrhythmic drug, such as esmolol, while a smaller percentage of patients also received levosimendan to increase the sensitivity of contractile proteins to calcium by blocking cTN. In all cases, it was found that not only was myocardial contractility increased without affecting abdominal relaxation, but patients also maintained a better ejection fraction, which was initially impaired (p < 0.001). Length of stay in the ICU, stay under sedation, and mechanical respiratory support were significantly higher in patients with MI (p < 0.001). Mortality was also 72% in these patients compared to the control group, in which mortality was about 21% (p < 0.001) (Table 3).

**Table 3:** Postoperative outcomes +Pearson’s x2 test ‡Student’s t-test ‡‡Mann-Whitney test IQR: interquartile range

		Total sample (N=105, 100%)	Myocardial ischemia			P
		Ν (%)	No (N=71; 67.6%)		Yes (N=34; 32.4%)	
Length of stay in hospital (days), SD (IQR)		27.9 (18.4), 25 (15 ─ 35)	24.4 (17.6), 18 (14 ─ 30)	35.3 (18), 32.5 (25 ─ 45)		0.001‡‡
Maximum dose of vasoconstrictor drugs, SD (IQR)		24.4 (21.6), 19 (10 ─ 35)	17.1 (18.5), 10 (5.5 ─ 20)	38.1 (20.5), 35 (20 ─ 50)		<0.001‡‡
Length of stay in the ICU (days), SD (IQR)		13.8 (14.9), 9 (4 ─ 17)	10.5 (13.3), 5 (3 ─ 11)	20.7 (15.9), 17.5 (10 ─ 25)		<0.001‡‡
Time of sedation, SD (IQR)		9.6 (13.7), 4.5 (1 ─ 12)	6 (10.7), 2 (1 ─ 6)	17.1 (16.1), 11.5 (7 ─ 22)		<0.001‡‡
Time of Mechanical Ventilation (days), SD (IQR)		9.9 (13.7), 5 (1.5 ─ 12.5)	6.6 (11.1), 2.5 (1 ─ 7)	16.8 (16.1), 12.3 (7 ─ 21)		<0.001‡‡
Outcome	Exit of ICU	83 (79)	65 (78.3)	18 (21.7)		<0.001+
	Mortality	22 (21)	6 (27.3)	16 (72.7)		

The mean ejection fraction was significantly lower postoperatively in patients with MI and showed a significant change (p < 0.001). In addition, patients who received antiplatelet therapy preoperatively had 52.5% MI (p = 0.001). The timing of the start of antiplatelet/anticoagulant therapy after surgery was similar in both groups. There also appeared to be no statistical correlation in postoperative antiplatelet/anticoagulant treatment. Of the drugs administered, acetylsalicylic acid (Salospir) and acenocoumarin (Sintrom) were used most frequently preoperatively. The rate of MI was significantly higher in patients with postoperative ECG abnormalities, with a predominant ECG finding of depression ST segmental interruption.

**Figure 2:**
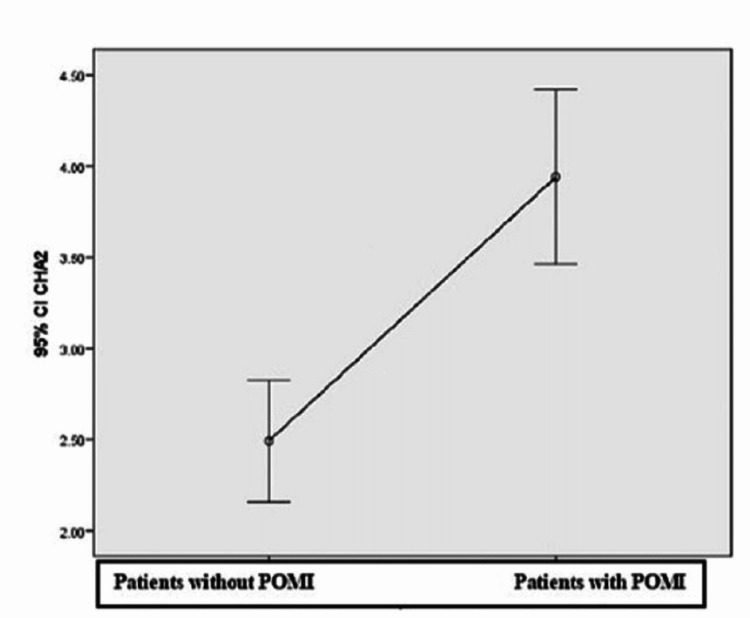
CHA2DS2-VASc score among the two groups CHA2DS2-VASc: congestive heart failure, hypertension, age ≥ 75 years, diabetes mellitus, stroke or transient ischemic attack (TIA), vascular disease, age 65 to 74 years, sex category

In all patients, the CHA2DS2-VASc score was also calculated to determine whether the postoperative risk of thrombosis could be predicted by the corresponding score [6]. This showed that 59.5% of patients who had an increased risk of thrombosis preoperatively had a POMI in the first three days (p < 0.001) (Figure 2).

The SOFA system was used to assess patient severity and was calculated daily based on the worst scores of the last 24 hours. After the initial assessment of the patients, the SOFA scale showed that patients who had the highest score at the time of admission to the ICU also had a higher mortality rate.

**Figure 3:**
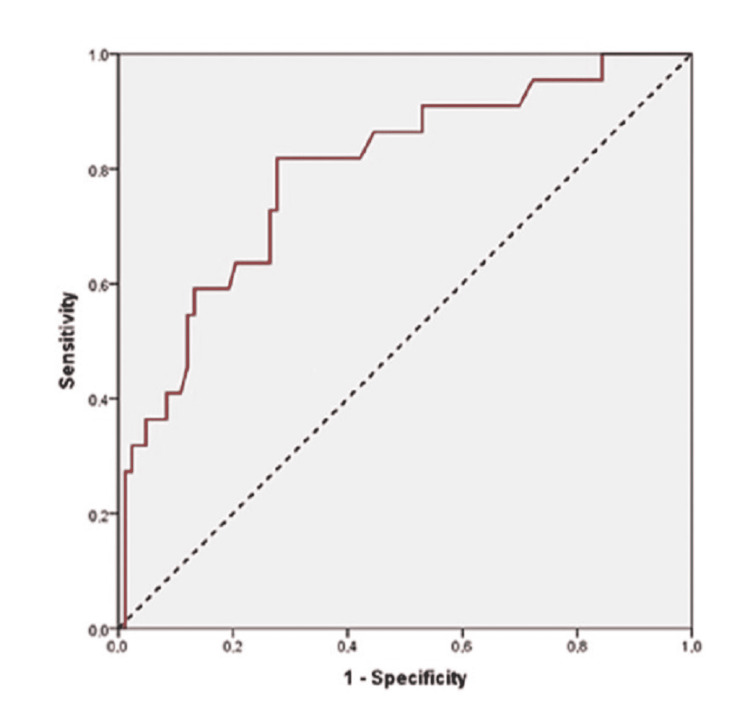
Receiving operating characteristic curve of preoperative troponin for predicting myocardial ischemia

A ROC analysis was performed to find the ideal cut-off value for preoperative troponin to predict MI (Figure 3). It was found that a preoperative value of 17.2 pg/ml and above was significantly associated with MI, with a sensitivity of 81.8% and a specificity of 72.3% (positive predictive value (PPV) = 43.9%; negative predictive value (NPV) = 93.8%).

Multivariate analysis showed that patients with dyslipidemia had an almost four-fold higher risk of MI. Patients with a troponin level of at least 17.2 pg/ml before surgery were more than four times as likely to develop MI. In addition, a higher CHA2DS2-VASc score was significantly associated with a higher likelihood of MI (Table 4).

**Table 4:** Multivariate logistic regression with myocardial ischemia as the dependent variable +Odds Ratio (95% Confidence Interval) CHA2DS2-VASc: congestive heart failure, hypertension, age ≥ 75 years, diabetes mellitus, stroke or transient ischemic attack (TIA), vascular disease, age 65 to 74 years, sex category

		OR (95% CI)+	Ρ
Dyslipidemia	Νο (reference)		
	Yes	3.95 (1.18 ─ 13.24)	0.026
Pre-op troponin	<17.2 (reference)		
	≥17.2	4.62 (1.59 ─ 13.42)	0.005
CHA2DS2-Vasc score		1.89 (1.31 – 2.72)	0.001

## Discussion

Most important results

**Figure 4:**
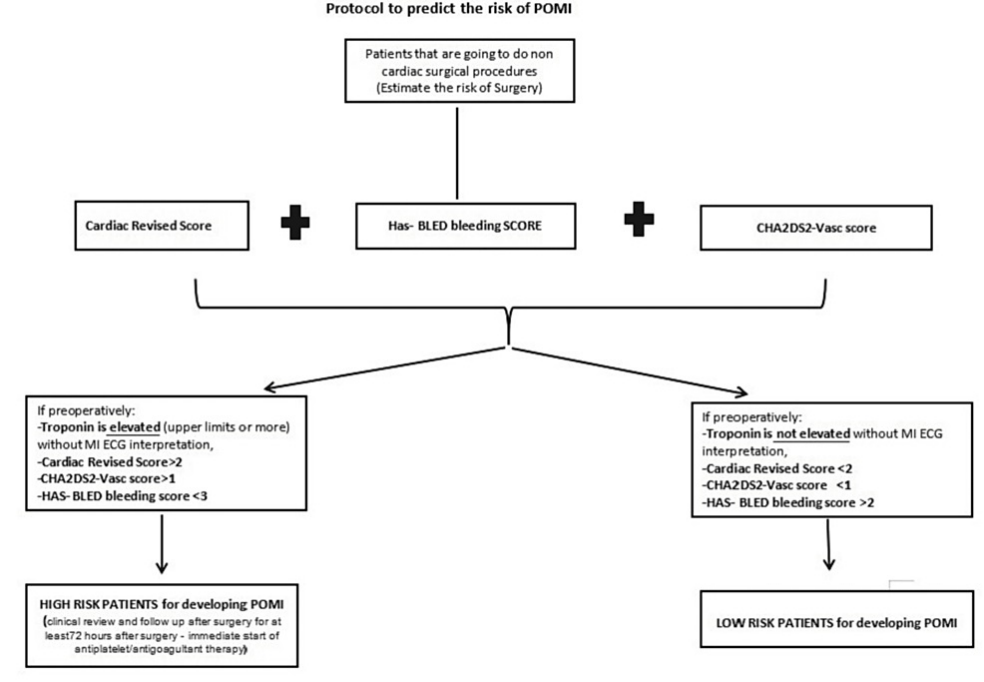
Protocol to predict the risk of POMI POMI: postoperative myocardial ischemia

In this study of 105 patients with a mean age of 69 undergoing non-cardiac surgery, we found that a preoperative troponin level with a high sensitivity of 17.2 pg/ml or higher was a prognostic factor for POMI. This criterion does not necessarily require the presence of an ischaemic feature. A higher risk of POMI was associated with increasing age, surgical procedures, and preoperative concomitant cardiovascular disease. Perioperative MI was associated with short- and long-term mortality 30 days after surgery. The CHA2DS2-VASc score for all patients before surgery was an independent predictor of risk, suggesting that measures reflecting a combination of risk factors, specific cardiac biomarkers, and cardiac scores may help predict POMI and mortality. We have developed a protocol to predict the risk of POMI (Figure 4). We propose to apply this model to all patients undergoing major non-cardiac surgery to assess the risk of developing POMI.

In a previous study (VISION; Vascular Events in Noncardiac Surgery Patients Cohort Evaluation), measurement of peak troponin in the first three days after non-cardiac surgery was an independent predictor of 30-day mortality [7]. Multivariable analysis of data from the Perioperative Ischemic Evaluation Trial (an international randomized controlled trial of 8,351 patients), adjusted for preoperative factors and perioperative complications, showed that elevations of a cardiac biomarker or enzyme such as troponin were an independent predictor of 30-day mortality in patients without an ischaemic feature [8]. While these results support our findings that elevated troponin levels after surgery increase short-term mortality, the many different methods of calculating troponin mean that the data are difficult to compare. Most studies of non-cardiac surgery investigating cardiac complications have focused on perioperative MIs rather than those in the period before surgery. The current study adds crucial new information by focusing on pre-surgery troponin elevations, classified as a consequence of MI, and considering possible confounding by pre-surgery risk factors. This is the first study to assess prognostic diagnostic criteria before surgery using specific cardiac biomarkers and comorbidities. Our results suggest that focusing on this period would miss prognostically relevant perioperative myocardial ischaemic events. The American Heart Association guidelines conclude that daily measurement of postoperative troponin levels during surgery is not useful [9]. Other studies find that a significant percentage of myocardial injuries are not detected. Therefore, the Canadian Cardiovascular Society guidelines for perioperative cardiac risk assessment and management in patients undergoing non-cardiac surgery recommend ‘determination of troponin in the first 72 hours after surgery in high-risk patients [10-11].

In addition, some studies have shown that troponin levels are sometimes elevated in surgical patients in the preoperative period. The risk of death was related to the timing of peak cTn. A higher preoperative cTn level was associated with higher postoperative mortality [12-14]. However, no studies have looked at preoperative troponin levels and their impact on the presence of POMI and mortality. In our protocol, we focused on the predictive value of troponin before surgery. Troponin and the revised cardiac risk index can be used to identify patients at high risk of POMI. We assume that patients with a score > 2 are at high cardiac risk. Combined with the CHA2DS2-Vasc score, this would narrow the percentage of patients likely to develop POMI. We could set the cut-off point for both investigations using all these criteria combined with high-sensitivity troponin before surgery. This could allow potentially immediate preventive measures to be taken throughout the perioperative period. Our predictive model of MI after surgery could be a simple tool for patients at intermediate and high risk of this ischaemic complication and guide clinical practice in surgical patients. Our pre-surgery risk score protocol is easy to use and suitable for non-cardiac surgery patients. However, further research is needed before implementation.

The costs associated with a troponin T monitoring program were moderate [15]. There is no doubt that the relevance of these studies could help physicians in their daily clinical practice, but there is a need for additional diagnostic tests with additional costs. Many studies suggest that surveillance consisting of troponin T measurements on the first day after surgery and postoperative days 1, 2, and 3 results in the lowest cost per MI detected. An economic analysis of perioperative troponin monitoring after non-cardiac surgery found that the 30-day rate of cardiovascular mortality and MI was reduced by 25% after initiation of treatment with acetylsalicylic acid and statins in patients with positive screening results [16-18]. In our study, the cost of troponin screening was not calculated. Still, it is not particularly cost-effective because it is measured in the first days in the ICU after surgery.

Limitations

This study was limited mainly by being carried out in a single center. External validation would be required to ensure its generalisability. Therefore, further studies with appropriate design are needed for future research to explore these issues and obtain other results.

## Conclusions

In patients at low risk, troponin testing should not be performed in the final postoperative period. In contrast, troponin levels in the first 72 hours should be considered in patients at relatively high risk. In addition, all patients should receive all available preventive measures during the intraoperative and postoperative periods. In countries where troponin measurement is not fully functional, this predictive model with the cardiac score, CHA2DS2-VASc score, and troponin levels could also be used as a prediction tool for POMI.
